# Biological Activities and Phenolic Profile of *Bursera microphylla* A. Gray: Study of the Magdalena Ecotype

**DOI:** 10.3390/plants14213357

**Published:** 2025-11-02

**Authors:** Heriberto Torres-Moreno, Julio César López-Romero, Max Vidal-Gutiérrez, Karen Lillian Rodríguez-Martínez, Ramón E. Robles Zepeda, Wagner Vilegas, Ailyn Oros-Morales

**Affiliations:** 1 Department of Chemical, Biological, and Agricultural Sciences, University of Sonora, Caborca 83600, Mexico; a218201507@unison.mx; 2 Department of Chemical, Biological, and Agricultural Sciences, University of Sonora, Navojoa 85880, Mexico; max.vidal@unison.mx; 3 Hermosillo Academic Unit, Sonora State University, Hermosillo 83100, Mexico; karen.rodriguez@ues.mx; 4 Department of Chemical and Biological Sciences, University of Sonora, Hermosillo 83000, Mexico; robles.zepeda@unison.mx; 5 Faculty of Pharmaceutical Sciences, Araraquara Campus, Paulista State University, Araraquara 14800-90, São Paulo, Brazil; vilegasw@gmail.com

**Keywords:** ecotypes, pharmacological properties, *Bursera*

## Abstract

*Bursera microphylla* A. Gray (Burseraceae) is a medicinal plant native to Sonora, Mexico, with antioxidant, anti-inflammatory, and antiproliferative properties. However, the pharmacological potential of its ecotypes remains underexplored. This study evaluated the biological activity and chemical composition of ethanolic extracts from the fruit and stem of the Magdalena ecotype. Total phenolic content was quantified using the Folin–Ciocalteu method, and phenolic profiles were characterized by ESI-IT-MS. Antioxidant activity was assessed by DPPH and FRAP assays; anti-inflammatory activity was evaluated by measuring nitric oxide (NO) and tumor necrosis factor-alpha (TNF-α) levels in LPS-activated RAW 264.7 macrophages. Antiproliferative activity was tested against LS180, C-33 A, and ARPE-19 cell lines using the MTT assay. Fruit extract exhibited higher phenolic content (180.6 ± 22.0 mg GAE/g) and ferric-reducing power (FRAP = 2034.3 ± 89.7 μM Fe(II)/g), whereas the stem extract showed stronger DPPH scavenging capacity (IC_50_ = 52.9 ± 0.02 μg/mL). For the first time, gallic acid glucoside, kaempferol rhamnoside, quercetin rhamnoside, and isorhamentin xyloside were identified in *B. microphylla* fruit extract. Furthermore, the fruit extract significantly reduced NO production (93.6 ± 4.6 μg/mL) and TNF-α levels (IC_50_ = 101.5 ± 9.1 μg/mL). It also showed strong cytotoxicity against C-33 A (IC_50_ = 0.6 ± 0.07 μg/mL) and LS180 (0.7 ± 0.01 μg/mL), with lower cytotoxicity in ARPE-19 cells (77.9 ± 4.3 μg/mL). These findings highlight the therapeutic potential of the Magdalena ecotype, likely associated with its phenolic and other bioactive metabolites that require further investigation.

## 1. Introduction

Traditional medicine has been used since ancient times to treat various health conditions. This includes plant-based therapies aimed at preventing or managing diseases [1]. Plant components such as leaves, stems, fruits, roots, seeds, and exudates are used either through direct consumption or through the preparation of infusions, macerates, and ointments, among others [2]. This ancient knowledge has served as a foundation for the pharmaceutical industry, as some drugs used today are obtained from plants or are synthetic derivatives of chemical compounds isolated from them [3]. This has supported the use of natural products as adjuvants in treating different diseases [4].

It is estimated that 80% of the world’s population continues to use plants as a primary treatment for various health problems, and they remain an important alternative for medical care in different regions of the world, such as Latin America, Africa, and Asia [5,6]. In this context, the World Health Organization (WHO) has promoted policies to explore ways to integrate traditional medicine into official health systems, viewing it as a viable option for primary health care [7]. This demonstrates that plants continue to represent a potential alternative for drug development. Therefore, it is important to continue investigating these sources.

Mexico has the fourth-greatest plant diversity in the world, with over 20,000 species recorded, approximately 50% of which are classified as endemic. Furthermore, it is important to highlight that the diversity of ecosystems and the variable climatic conditions can affect the characteristics and composition of these plants, including their medicinal properties [8,9]. Approximately 15% of Mexican plants are used in folk medicine to treat various health conditions by consuming their constituents [10]. However, only 5% of these plants have been studied [8]. Based on the above, it seems feasible to continue analyzing Mexican medicinal plants to study their pharmacological properties. This analysis could determine the feasibility of using these plants to extract chemical compounds with pharmacological effects.

*Bursera microphylla* A. Gray (Burseraceae) is a plant native to the Sonoran Desert, known as “torote blanco”, and used in traditional medicine to treat several health conditions, including wound healing, headaches, throat conditions, and viral infections, through the use of its different components, such as resins, flowers, leaves, fruits, and bark, however, the presence of some constituents, such as leaves and fruit plants, varies depending on the harvest season [11,12]. In the Sonoran State, *B. microphylla* is distributed in Cajeme, Hermosillo, Magdalena, and Caborca [12,13]. The extracts and bioactive compounds (lignans and terpenoids) of the resin of *B. microphylla* ecotype Hermosillo exhibited antiproliferative potential against cancer cells [12,13]. Previous studies have also shown that extracts from the stems, leaves, and fruit of *B. microphylla,* specifically the Caborca ecotype, exhibit antioxidant, anti-inflammatory, and antiproliferative potential against cancer cells, which is attributed to the presence of phenolic compounds and lignans [14,15]. Additionally, it is well known that the geographical location of the plants plays a crucial role in their biological activity and the types of bioactive compounds produced, as this is influenced by biotic and abiotic factors that induce their biosynthesis [16,17]. Since *B. microphylla* is a plant distributed throughout the Sonoran Desert and Sonora is the second-largest state in Mexico, where weather variations are common throughout its territory [18,19], it seems relevant to analyze the effect of ecotypes on the biological activities and the profile of phenolic compounds present in *B. microphylla*.

This study aimed to evaluate the antioxidant, antiproliferative, and anti-inflammatory properties, as well as the phenolic profile, of the *B. microphylla* ecotype from Magdalena.

## 2. Results

### 2.1. Phenolic Content and Profile

Total phenolic content, considered a marker of antioxidant potential, was quantified in the fruit and stem extracts using the Folin–Ciocalteu method. The fruit extract of the *B. microphylla* Magdalena ecotype exhibited a significantly higher concentration (*p* < 0.05) (180.6 ± 22.02 mg GAE/g) compared to the stem extract (73.05 ± 0.52 mg GAE/g).

**Figure 1:**
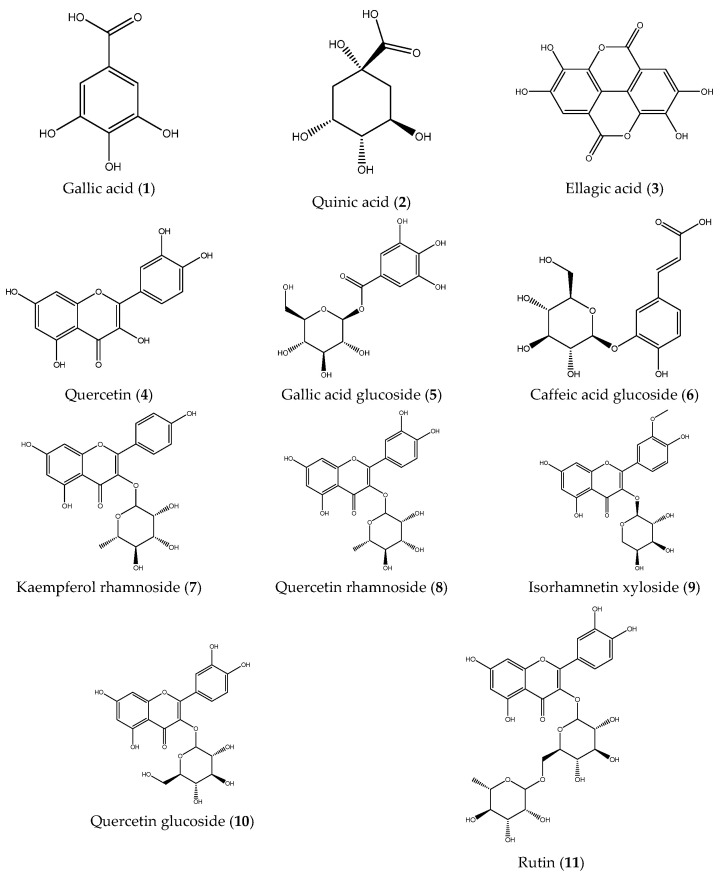
Phenolic compounds identified in the *Bursera microphylla* Magdalena ecotype.

Subsequently, the phenolic composition of the extracts was characterized by ESI-IT-MS^n^. Fruit and stem extracts were analyzed in negative ionization mode, and fragmentation patterns were examined to elucidate the structures of the compounds (Table 1). A total of eleven compounds were identified: gallic acid (1), quinic acid (2), ellagic acid (3), quercetin (4), gallic acid glucoside (5), caffeic acid glucoside (6), kaempferol rhamnoside (7), quercetin rhamnoside (8), isorhamentin xyloside (9), quercetin glucoside (10), and rutin (11). Notably, compounds **6**, **7**, **8**, and **9** were identified for the first time in *B. microphylla* (Figure 1). As can be observed in the full scans (Figures S1 and S2), the compounds identified in the fruits of *B. microphylla* are found in a very homogeneous relative abundance among themselves, compared to the compounds identified in the stems, where the most abundant compound is quinic acid (*m*/*z* 191 [M-H]-), followed by caffeinic acid glucoside (*m*/*z* 341 [M-H]-).

### 2.2. Antioxidant Activity

Since the fruit and stem extracts of *B. microphylla* Magdalena ecotype were identified as promising sources of phenolic compounds, the antioxidant capacity was evaluated using two complementary assays, DPPH and FRAP (Table 2). The DPPH assay measures free radical scavenging capacity, whereas the FRAP assay evaluates ferric-reducing potential. The results showed that both extracts exhibited distinct antioxidant behaviors: the stem extract was 1.9 times more effective in stabilizing the DPPH radical compared to the fruit extract (*p* < 0.05). In contrast, the fruit extract showed 1.4 times higher reducing capacity in the FRAP assay compared to the stem extract (*p* < 0.05).

### 2.3. Anti-Inflammatory Activity

**Figure 2:**
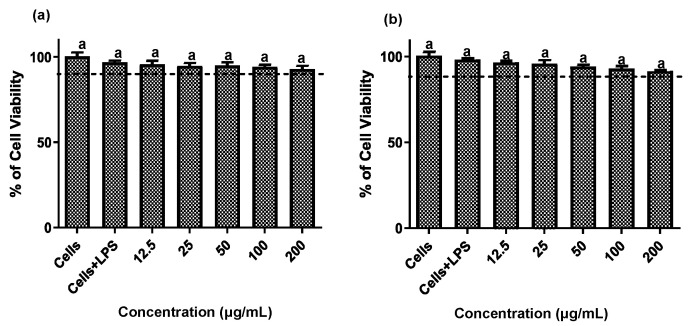
Cytotoxic effect of extracts of the *Bursera microphylla* Magdalena ecotype in the RAW 264.7 cell line. (**a**) Fruit extract, (**b**) stem extract. All values represent mean ± standard deviation (SD) of three independent experiments.

**Figure 3:**
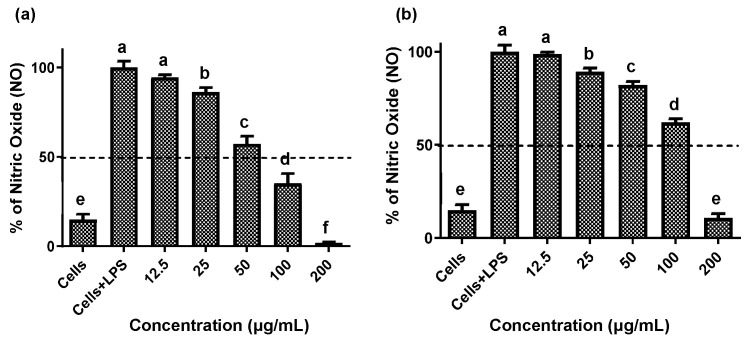
Effect of extracts of the *Bursera microphylla* Magdalena ecotype on NO production in the RAW 264.7 cell line. (**a**) Fruit extract, (**b**) stem extract. All values represent mean ± standard deviation (SD) of three independent experiments. ^a–f^ Bars with different letters indicate significant statistical differences (*p* < 0.05).

**Figure 4:**
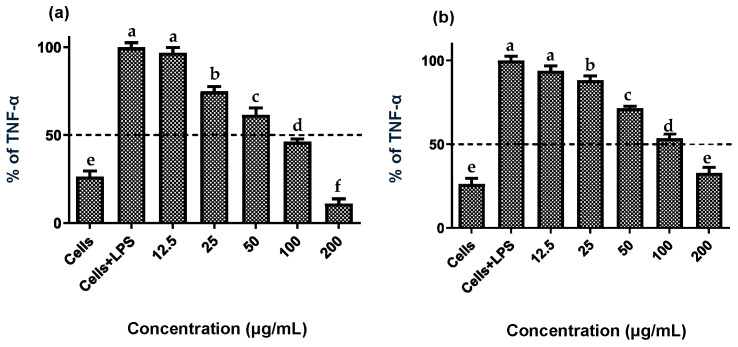
Effect of extracts of the *Bursera microphylla* Magdalena ecotype on TNF-α production in the RAW 264.7 cell line. (**a**) Fruit extract, (**b**) stem extract. All values represent mean ± standard deviation (SD) of three independent experiments. ^a–f^ Bars with different letters indicate significant statistical differences (*p* < 0.05).

Establishing non-cytotoxic concentrations of the extracts is a critical preliminary step before assessing their anti-inflammatory effects in cellular models. This ensures that any reduction in inflammatory markers is due to a specific modulation of the inflammatory process rather than cell death [20]. Both fruit and stem extracts showed no cytotoxicity toward RAW 264.7 cells, maintaining cell viability ≥ 90% (Figure 2). Moreover, both extracts effectively reduced NO and TNF-*α* levels in LPS-activated RAW 264.7 cells. As shown in Figure 3 and Figure 4, the LPS-activated cells exhibited significantly higher levels of NO and TNF-α compared to the basal control. Additionally, both extracts inhibited the production of NO and TNF-α in a dose-dependent manner. Importantly, the fruit extract exerted a stronger inhibitory effect than the stem extract on the levels of these inflammatory mediators. Furthermore, IC_50_ values calculated for both extracts (Table 3) confirmed that the fruit extract possesses a more potent anti-inflammatory activity than the stem extract.

### 2.4. Antiproliferative Activity

The antiproliferative activity of fruit and stem extracts of the *B. microphylla* Magdalena ecotype against various cell lines was evaluated using the MTT assay. Both extracts demonstrated the ability to inhibit the proliferation of the cancer cell lines LS180 and C-33 A, with greater activity observed against C-33 A. Moreover, the fruit extract showed stronger effects on LS180 cancer cells, exhibiting lower IC_50_ values compared to the stem extract (Table 4). In contrast, both extracts displayed higher IC_50_ values in the non-cancerous ARPE-19 cell line compared to cancer cells. The selectivity index (SI) indicated that both extracts selectively inhibited the proliferation of LS180 and C-33 A cancer cells (Table 5).

## 3. Discussion

The pharmacological properties of *B. microphylla* have been associated with bioactive metabolites such as phenolic compounds, lignans, and terpenoids [9,11,17]. Vidal-Gutiérrez et al. [15] established the antioxidant capacity of *Bursera microphylla* Caborca ecotype extracts, correlating this activity with the phenolic profile constituted by phenolic acids (gallic, quinic, and ellagic acids) and flavonoids (including quercetin, catechin, and kaempferol). In this study, the total phenolic content of fruit and stem extracts was determined by the Folin–Ciocalteu method. The fruit extract of the Magdalena ecotype showed higher total phenolic content than the stem extract (180.6 ± 22.0 mg GAE/g vs. 73.05 ± 0.52 mg GAE/g). By comparison, Vidal-Gutiérrez et al. [15] reported that the leaf extract of the Caborca ecotype revealed higher phenolic content than the fruit extract (243.4 ± 11.9 mg GAE/g vs. 104.3 ± 10.8 mg GAE/g).

For the large-scale production of pharmaceutical products, it is important to establish the conditions that favor the highest yield of bioactive metabolites in plants [21,22]. The concentration of phenolic compounds has been shown to increase in response to environmental factors such as UV radiation, temperature, low water availability, and the presence of pests [23]. Therefore, the higher concentration of phenolic compounds observed in fruit extracts of the *B. microphylla* Magdalena ecotype may be closely linked to its ecosystem. Since Magdalena is characterized by lower temperatures, this may be related to increased production of these compounds, as plants often produce phenolics as a defense against cold stress. Likewise, greater annual rainfall can enhance nutrient availability in the soil, further promoting the production of phenolic compounds. In addition, specific developmental stages, such as flowering or fruit ripening, are also associated with especially high levels of these compounds [24,25].

ESI-IT-MSn analysis of fruit and stem extracts of the Magdalena ecotype allowed the identification of 11 phenolic compounds, of which caffeic acid glucoside (6), kaempferol rhamnoside (7), quercetin rhamnoside (8), and isorhamentin xyloside (9) were identified for the first time in *B. microphylla*. Compound **1** showed a molecular ion at *m*/*z* 169 [M-H]- in MS^1^, followed by MS^2^ fragmentation yielding a fragment ion at *m*/*z* 125 [M-H]-, and finally a fragment at *m*/*z* 97 [M-H]- in MS^3^, a fragmentation pattern consistent with gallic acid [26,27]. Compound **2** displayed a molecular ion at *m*/*z* 191 [M-H]- in MS^1^; when fragmentation was performed by MS^2^, the molecular ions at: *m*/*z* 173 [M-H]-, *m*/*z* 127 [M-H]-, *m*/*z* 111 [M-H]-, *m*/*z* 93 [M-H]-, *m*/*z* 85 [M-H]- were produced, a fragmentation pattern consistent with the presence of quinic acid [28].

Compound **3** yielded a molecular ion at MS^1^ of *m*/*z* 301 [M-H]-, at MS^2^, the fragments *m*/*z* 283 [M-H]-, *m*/*z* 271 [M-H]-, and *m*/*z* 257 [M-H]- were obtained, a pattern that coincides with the presence of ellagic acid [26]. Compound **4** presented a molecular ion at *m*/*z* 301 [M-H]- and by MS^2^, the products *m*/*z* 179[M-H]- and *m*/*z* 151 [M-H]- were obtained; these fragments coincide with the fragmentation pattern of the compound quercetin [29,30]. Compound **5** presented a molecular ion at *m*/*z* 331 [M-H]- by MS^1^, followed by an ion at *m*/*z* 169 [M-H]- by MS^2^ and an ion at *m*/*z* 125 [M-H]- by MS^3^, which are associated with the compound gallic acid glycoside [26,31]. Compound **6** showed a molecular ion at *m*/*z* 341 [M-H]- by MS^1^, followed by fragment *m*/*z* 179 [M-H]- by MS^2^, and with MS^3^, a fragment at *m*/*z* 135 [M-H]-; this pattern matches the compound caffeic acid glucoside [31].

Compound **7** showed a molecular ion at *m*/*z* 431 [M-H]- by MS^1^, followed by an ion at *m*/*z* 285 [M-H]- by MS^2^, a fragmentation pattern consistent with the compound kaempferol rhamnoside [27]. Compound **8** presented a molecular ion at *m*/*z* 447 [M-H]- by MS^1^, then with MS^2^, a molecular ion at *m*/*z* 301 [M-H]-, and by MS^3^, the molecular ions at *m*/*z* 179 [M-H]- and at *m*/*z* 151 [M-H]-, indicating the presence of the compound quercetin rhamnoside [27]. Compound **9** presented a molecular ion by MS^1^ at *m*/*z* 447 [M-H]-, and by MS^2^, the products obtained were molecular ions at *m*/*z* 315[M-H]-, *m*/*z* 301[M-H]-, *m*/*z* 131 [M-H]-, *m*/*z* 161 [M-H]-, *m*/*z* 285 [M-H]- and at *m*/*z* 379 [M-H]-, indicating the presence of isorhamentin xyloside [27,32].

Compound **10** showed a molecular ion at *m*/*z* 463 [M-H]- by MS^1^, an ion at *m*/*z* 301 [M-H]- by MS^2^, and molecular ions at *m*/*z* 179 [M-H]- and *m*/*z* 151 [M-H]- by MS^3^. These patterns are consistent with the compound quercetin glycoside [27,32]. Compound **11** presented a molecular ion at *m*/*z* 609 [M-H]- by MS^1^, at *m*/*z* 301 [M-H]- by MS^2^ and the products at *m*/*z* 179 [M-H]- and at *m*/*z* 151 [M-H]- by MS^3^, characteristic fragmentation of the compound rutin [28,32].

The evaluation of antioxidant activity revealed that both extracts (from fruit and stem) of the *B. microphylla* Magdalena ecotype possess the ability to neutralize free radicals and reduce metals. This was established through the DPPH and FRAP assays, which confirmed that the phenolic compounds present in these extracts contribute significantly to their antioxidant capacity.

Vidal-Gutiérrez et al. [15] investigated the antioxidant potential of *Bursera microphylla* ecotype Caborca and attributed this activity to its phenolic profile, including phenolic acids such as gallic, quinic, and ellagic acids, as well as flavonoids like quercetin, catechin, and kaempferol. The leaf extract in that study exhibited a higher antioxidant activity (DPPH IC_50_ = 34.7 ± 1.8 µg/mL) compared to the fruit extract (121.6 ± 1.2 µg/mL). These findings underscore the greater radical scavenging capacity of the leaf extract of the Caborca ecotype. Nevertheless, when comparing the activity of fruit extract from both ecotypes, the Magdalena ecotype is more effective at eliminating free radicals, as demonstrated by its lower DPPH IC_50_ value (105.40 ± 0.48 µg/mL). According to the classification of antioxidant activity by Blois [33], the stem extract of the Magdalena ecotype falls into the strong antioxidant category, while the fruit extract is categorized as moderate.

The FRAP assay revealed that the fruit extract of the Magdalena ecotype exhibited greater ferric reducing activity (2034.3 ± 89.7 µM Fe(II)/g) compared to the stem extract (115.3 µM Fe(II)/g). Based on Wong et al. (2006) criteria [34], extracts with FRAP values above 500 µM Fe(II)/g are considered to have high reducing power. Both Magdalena extracts fall into this category, confirming their strong electron-donating capacity. In comparison, the B. microphylla Caborca ecotype showed even higher activity in leaves and fruit extracts (3809.4 ± 242 and 1168.6 ± 101.3 µM Fe(II)/g, respectively) [15], suggesting that Caborca leaf extract has superior ferric-reducing potential.

Differences in antioxidant activity between fruit and stem extracts can be attributed to their distinct functional roles and chemical profiles. Fruits often accumulate a higher quantity of phenolic compounds—such as flavonoids and polyphenols—compared to stems, as these metabolites provide critical protection against oxidative stress and help preserve seed viability and facilitate dispersal [35]. However, it is not just the total phenolic content but the specific types of phenolic compounds that determine antioxidant potency, since molecules like anthocyanins, flavonols, and phenolic acids exhibit differing radical-scavenging and metal-reducing capabilities [36].

Phenolic compounds are characterized by the presence of one or more hydroxyl groups directly attached to one or more aromatic rings [36,37]. The antioxidant function of these compounds is due to different mechanisms. For example, ellagic acid, present in the fruit and stem extracts of the Magdalena ecotype, in addition to stabilizing free radicals by donating electrons, can bind to metal ions, such as iron. By chelating these metals, it can prevent the generation of free radicals. Likewise, another compound that meets the same characteristics is gallic acid, present in the fruit extract of the Magdalena ecotype [38]. On the other hand, flavonoids are made up of two benzene rings (A and B) joined by a pyran ring (C). The flavonoid compound rutin present in the stem extract is characterized by stabilizing free radicals through hydrogen transfer and electron transfer, which gives it a high stabilizing and antioxidant potential, because the electroactive site with the greatest capacity to trap free OH- radicals is in the C3’ and C4’ site of the B ring. While the flavonoid quercetin present in both extracts demonstrates an antioxidant potential due to its ability to react with metal ions and free radicals, the antioxidant activity lies in the presence of 5 OH groups, present in C3’ and C4’ of the B ring, C3 of the C ring and C5 and C7 of the A ring [37,39,40].

This study demonstrated that fruit and stem extracts of the *B. microphylla* Magdalena ecotype reduced NO and TNF-α production in LPS-activated RAW 264.7 macrophages. Torres-Moreno et al. [14] similarly assessed the anti-inflammatory efficacy of fruit, stem, and leaf extracts from the ecotype Caborca across the four seasons of the year. They found that seasonality modulates this activity: fruit extract IC_50_ values were 131 ± 5.6 µg/mL (spring) and 153.4 ± 6.8 µg/mL (summer), while leaf extracts ranged from 183.3 ± 24.4 µg/mL in spring to 195.5 ± 9.7 µg/mL in winter, and stem extracts ranged from 152.4 ± 8.3 µg/mL in spring to 175.5 ± 13.8 µg/mL in winter. By comparison, the extracts from the Magdalena ecotype demonstrated greater anti-inflammatory potency, with IC_50_ values of 93.6 ± 4.6 µg/mL for the fruit extracts and 119.7 ± 2.8 µg/mL for the stem extracts, lower values than those seen in the corresponding Caborca extracts, highlighting the significant influence of geographic origin and environmental conditions on metabolite bioactivity.

Phenolic compounds are known to exert anti-inflammatory effects through multiple mechanisms. For example, quercetin and kaempferol have been shown to reduce NO production in the RAW 264.7 cells by inhibiting the overexpression of iNOS and COX-2, in part by blocking the NF-κB signaling pathway. Similarly, ellagic acid and gallic acid (found in *B. microphylla*) can interfere with TNF-α production and release, suppressing inflammatory pathways such as TLR4/NF-κB activation [41,42].

During inflammation, TNF-α secretion by macrophages intensifies oxidative stress and activates NF-κB, which in turn upregulates iNOS expression and further elevates NO level [43,44]. While phenolic compounds appear to play a key role in the anti-inflammatory effects observed in the Magdalena ecotype extracts, future bioassay-guided studies are needed to isolate and verify the specific active metabolites, whether phenolics like quercetin, ellagic, or gallic acids, or other bioactives such as terpenes or lignans are involved.

There is a direct relationship between chronic inflammation and cancer. Cytokine production during inflammation triggers the release of ROS and RNS by macrophages. These molecules can cause DNA damage in cells [45]. The NO radical has two signaling pathways, one of which is dependent on soluble guanylate cyclase (sGC), with the subsequent generation of cyclic guanosine monophosphate (cGMP). The other pathway is known as the oxidative pathway. In the sGC-independent radical, NO reacts with the active site of sGC and produces cGMP; cGMP activates cyclic nucleotide-dependent protein kinases, which phosphorylate different protein substrates related to the metastatic potential of tumor cells [46]. On the other hand, the oxidative pathway can cause post-translational modifications in proteins by inducing alterations in DNA. For example, some of the factors that can change transcription are NF-κB [47]. The NO radical is also capable of interacting with molecular oxygen or the superoxide radical, forming more reactive species such as nitrous oxide (N_2_O_3_) or peroxynitrite (ONOO^−^). These molecules ultimately activate more free radicals capable of interacting with the DNA as mutagens, contributing to the induction and progression of cancer [48].

In addition to having the ability to stabilize free radicals, phenolic compounds are capable of inducing mechanisms that activate cell death in cancer cells and inhibit cell proliferation [49]. In a study published by Vidal-Gutiérrez et al. [50], three species of the genus *Bursera*, *B. laxifrola*, *B. microphylla*, and *B. hindsiana*, were studied. The methanolic extract of the bark of *B. laxiflora* demonstrated selective antiproliferative activity in cancer cell lines. However, *B. microphylla* resin showed the best antiproliferative activity against HeLa (human cervical cancer), A549 (human alveolar carcinoma), and M12A.C3.F6 (murine B-cell lymphoma). The HeLa cell line was the most sensitive in that study (IC_50_ = 13.8 μg/mL). Likewise, extracts of fruit, stem, and leaves of *B. microphylla* Caborca ecotype were evaluated for antiproliferative activity against the cancer cell lines C-33 A (cervical cancer cell line), HeLa, and A549. The results obtained demonstrated that the cancer cell lines were effectively inhibited by the treatments, with the fruit extracts showing the best activity [14]. The fruit extract of the Magdalena ecotype exhibited antiproliferative activity on the C-33 A cervical cancer cell line (IC_50_ = 0.6 ± 0.07 µg/mL), closely matching the Caborca ecotype fruit extract (IC_50_ = 0.8 ± 0.01 µg/mL). Both extracts showed similar behavior on the non-cancerous ARPE 19 cells, with IC_50_ values of 77.9 ± 4.3 µg/mL for the Magdalena ecotype and 75.6 ± 7.6 µg/mL for the Caborca ecotype. These results underscore the comparable antiproliferative activity against C-33 A cancer cells and consistent selectivity against non-cancerous ARPE 19 cells.

Likewise, lignan and terpene compounds have been reported to exhibit antiproliferative and anti-inflammatory activity in extracts of *B. microphylla* [12,13], indicating that they may act synergistically with phenolic compounds, enhancing their biological potential. This suggests the future study of other compounds, such as lignans and terpenes from the *B. microphylla* Magdalena ecotype.

Overall, variations in the biological activity spectra of the different ecotypes and constituents of *B. microphylla* are evident. However, comparing extracts of a similar nature (fruit extract) from the different *B. Microphylla* ecotypes show that the Magdalena extract exhibited an increase in the biological activity profile (antioxidant, anti-inflammatory, and antiproliferative), which can be related to the concentration and profile of bioactive compounds, such as phenolics, that increased in this extract. These changes in the concentration of bioactive compounds can be associated with environmental and growth conditions, as explained previously. Additionally, in the Magdalena ecotype, the fruit extract increased the biological potential compared to the stem extract. This behavior could be associated with a higher concentration and greater number of identified phenolic compounds, which have demonstrated a broad spectrum of biological activities. However, it is important to mention that other bioactive compounds may be present in the analyzed extracts.

## 4. Materials and Methods

### 4.1. Plant Collection and Extracts Preparation

*Bursera microphylla* A. Gray (Figure S3) was collected in Magdalena, Sonora, during the spring of 2018 (30°37′07.8″ N 110°56′43.0″ W) and identified by Engineer Jesús Sánchez Escalante, chief of the University of Sonora Herbarium. The stems and fruits of ten specimens were separated, crushed, and macerated with ethanol in a 1:10 ratio (*w*/*v*) at room temperature for 10 days, with occasional stirring. The extracts were filtered, and the solvent was evaporated at 45 °C under reduced pressure using a rotary evaporator. The extracts obtained from fruit (EFM) and stem (ETM) were stored at −20 °C until use [15].

### 4.2. Total Phenolic Content

The total phenolic content was measured in the EFM and ETM using the Folin–Ciocalteu method. Briefly, 10 μL of the extracts were mixed with 80 μL of distilled water, 40 μL of 0.25 N Folin–Ciocalteu reagent, 60 μL of 5% sodium carbonate, and 80 μL of distilled water. The mixture was incubated in the dark for one hour, after which the absorbance was read at 750 nm. The results were expressed as mg of gallic acid equivalent (GAE/g) of dry sample [15].

### 4.3. ESI-IT-MS^n^ Analysis

Extracts were dissolved in a mixture of MeOH-H_2_O (85:15, *v*/*v*) for clean-up on a C18 solid-phase extraction cartridge (STRATA™ C18-E, Phenomenex, Torrance, CA, USA) that was eluted with a mobile phase consisting of MeOH-H_2_O (85:15, *v*/*v*). The resulting elutions were dried and individually dissolved in LC-MS (Liquid Chromatography-Mass Spectrometry) grade MeOH (methanol) and filtered through a 0.22 µm pore size PVDF (polyvinylidene fluoride) filter. The samples (5 µg/mL) were directly injected into a Thermo LTQ-XL apparatus coupled to an electrospray ionization (ESI) source and an ion trap analyzer (IT-MS^n^) in negative mode. Analyses were performed at a capillary voltage of 10 V and a flow of 30 arbitrary units of sheath gas. Fragmentations were performed by the collision-induced dissociation (CID) method with helium and a collision energy of 30 eV. The fragmentation pattern method was used to identify the compounds [15].

### 4.4. Antioxidant Activity

#### 4.4.1. DPPH Assay

The antioxidant activity of *B. microphylla* extracts was evaluated using the 1,1-diphenyl-2-picrylhydrazyl (DPPH) free radical scavenging assay. Briefly, 100 μL of EFM or ETM (31.25–250 μg/mL) was mixed with 100 μL of the DPPH solution (300 μM) and added to a 96-well microplate. Later, the microplate was incubated in darkness for 30 min and the absorbance was read at 517 nm in a microplate reader (iMARK microplate reader, BIO-RAD, Hercules, CA, USA). Finally, the average concentration of the extracts that inhibited 50% of the DPPH radical (IC_50_) was calculated by linear regression [15].

#### 4.4.2. FRAP Assay

The FRAP reagent was prepared with acetate (300 mM), TPTZ (40 mM dissolved in 40 mM HCl), and aqueous ferric chloride (20 mM) in a 10:1:1 ratio. Briefly, FRAP reagent (280 μL) was added to each extract (20 μL), and the reaction was incubated for 30 min in the absence of light. The plate was then read at 630 nm in a microplate reader. Results were expressed as micromoles (μM) of ferrous ion Fe(II)/g of dry sample [15].

### 4.5. Cell Culture

Cell lines were maintained in Dulbecco’s Modified Eagle Medium (DMEM), supplemented with 5% heat-inactivated fetal bovine serum (FBS) and 100 U/mL penicillin. Cultures were grown in 25 cm^2^ tissue culture flasks and incubated at 37 °C in a humidified atmosphere containing 5% CO_2_ and 95% relative humidity using an Isotherm incubator (Thermo Fisher Scientific, Waltham, MA, USA). The human cervical carcinoma cell line C-33 A was provided by Dr. Salomón Hernández Gutiérrez (School of Medicine, Panamerican University, Mexico City, Mexico). The LS180 human colon adenocarcinoma cell line was obtained from the American Type Culture Collection (ATCC, Rockville, MD, USA). The RAW 264.7 murine macrophage cell line, transformed by the Abelson murine leukemia virus, was kindly provided by Dr. Emil A. Unanue from the Department of Pathology and Immunology, Washington University School of Medicine, St. Louis, MO, USA [14].

### 4.6. Anti-Inflammatory Activity

#### 4.6.1. Cytotoxic Effect

The MTT assay was used to determine the cytotoxic effect of the extracts on the RAW 264.7 cell line. A cell suspension with a density of 5 × 10^5^ cells/mL was prepared from a cell culture with a confluence of ≥95%. Subsequently, 100 µL of the cell suspension was placed in a 96-well cell culture plate (Costar, Corning, NY, USA) and incubated for 24 h. After this time, the cells were treated for 24 h with an aliquot of medium (100 µL) containing the extracts (12.5–200 µg/mL). Subsequently, the supernatant was removed from the plate and washed with phosphate-buffered saline (PBS). Next, 90 μL of DMEM and 10 μL of MTT solution (5 mg/mL) were added to each well of the plate. The plate was incubated for 4 h, and the formazan crystals were resuspended in acidic isopropanol. Finally, absorbances at 570 and 630 nm were read in a microplate reader (iMARK microplate reader, BIO-RAD). The concentrations evaluated were considered cytotoxic when cell viability was <90% and was calculated with the following equation: Viability (%) = Abs sample/Abs control × 100 [14].

#### 4.6.2. Quantification of NO Production

The effect on NO production was evaluated in LPS (0111:B1)-activated RAW 264.7 cells using the Griess reaction. A cell suspension was prepared at a concentration of 5 × 10^5^ cells/mL. One hundred microliters of the cell suspension were then added to a 96-well plate, and the plate was incubated for 24 h. Cells were then stimulated with LPS (1 μg/mL) in the presence or absence of extracts at different concentrations (12.5–200 μg/mL) for 24 h. After this time, 100 μL of the supernatant was collected and combined with an equal volume of Griess reagent. After a 10 min incubation at room temperature and in darkness, the absorbance was measured at 540 nm using a microplate reader (iMARK microplate reader, BIO-RAD). The NO concentration was determined by comparison with a sodium nitrite standard curve [51]. The results were expressed as IC_50_ values (meaning the concentration that inhibits NO production by 50%) and were calculated by linear regression [14].

#### 4.6.3. Quantification of TNF-α Production

The effect of the extracts on TNF-α production was performed using a sandwich ELISA kit (Thermo Fischer Scientific, Waltham, MA, USA) according to the manufacturer’s instructions. The assay was performed by placing 5 × 10^5^ cells/mL in a 96-well plate (Costar, Corning, NY, USA). Subsequently, the cells were stimulated with LPS 0111:B1 (1 μg/mL) in the presence or absence of the extracts (12.5–200 μg/mL) for 24 h. TNF-α quantification was performed by adding the supernatant from the cells (50 μL) to microplates coated with antibodies specific for mouse TNF-α. The plate was then washed with 400 μL of wash buffer, and 50 μL of anti-TNF-α secondary antibodies labeled with peroxidase were added. After a second incubation of 2 h, a wash with 400 μL of wash buffer was performed to remove unbound antibody. Tetramethylbenzidine (TMB) (100 μL) was then added, and the plate was incubated for 30 min. Finally, the absorbance was read in a spectrophotometer (iMARK microplate reader, BIO-RAD) at 450 nm [51].

### 4.7. Antiproliferative Effect

#### 4.7.1. MTT Assay

The antiproliferative activity of *B. microphylla* extracts against LS180 (human colon cancer), C-33 A (human cervical cancer), and ARPE 19 (noncancerous retinal pigment epithelium) cell lines was evaluated by MTT. Briefly, 50 μL of a cell suspension (2 × 10^5^ cells/mL) was placed in a 96-well plate (Costar, Corning, NY, USA) and incubated for 24 h. The extracts were subsequently dissolved in DMSO and diluted in DMEM to achieve a final DMSO concentration of 0.5%, a level considered non-cytotoxic and not inhibitory to cell proliferation. The cells were then exposed to the extracts (0.2 to 3.2 μg/mL) for 48 h. Subsequently, formazan crystals were solubilized, and the absorbance was read at 570 and 630 nm in a microplate reader (iMARK microplate reader, BIO-RAD). Results are reported as IC_50_ values (meaning the concentration required to inhibit cell proliferation by 50%) and were calculated by linear regression [14].

#### 4.7.2. Selective Index

To establish the cytotoxic selectivity of *B. microphylla* Magdalena ecotype extracts toward cancer cells, the Selectivity Index (SI) was determined. The SI was calculated by dividing the IC_50_ value obtained in non-cancerous cells (ARPE-19) by the IC_50_ against each respective cancer cell line. The extract is considered selective for a cell line when the SI > 10 [14].

### 4.8. Statistical Analysis

Statistical analysis was conducted using IBM SPSS Statistics 20 software. Differences among treatment groups were evaluated using one-way analysis of variance (ANOVA), followed by Tukey’s test for multiple comparisons. Results are presented as the mean ± standard deviation (SD) from three independent experiments. A *p*-value of <0.05 was considered statistically significant [14].

## 5. Conclusions

Fruit and stem extracts from the *B. microphylla* Magdalena ecotype showed high antioxidant, anti-inflammatory, and antiproliferative capacities. These biological activities could be attributed to the presence of phenolic compounds. However, more in-depth studies are needed to understand the bioactive compounds associated with these biological activities. Chemical analysis identified, for the first time, the phenolic compounds gallic acid glucoside, kaempferol rhamnoside, quercetin rhamnoside, and isorhamentin xyloside in *B. microphylla*, contributing to our understanding of the plant’s phytochemistry. The results suggest that environmental conditions associated with the ecosystem modulate the biological activity of *B. microphylla*, underscoring the need to explore in future studies the biological activity and chemical composition of other *B. microphylla* ecotypes distributed in the diverse ecosystems of northwestern Mexico.

## Figures and Tables

**Table 1:** Identification of phenolic compounds by ESI-IT-MS^n^ in fruit and stem extracts of the *Bursera microphylla* Magdalena ecotype.

	Compound	MS^1^	MS^2^	MS^3^	Fruit	Stem
1	Gallic acid	169	125	97	**X**	
2	Quinic acid	191	173, 127, 111, 93, 85	-	**X**	
3	Ellagic acid	301	283, 272, 257	-	**X**	**X**
4	Quercetin	301	179, 151	-	**X**	**X**
5	Gallic acid glucoside	331	169	125	**X**	
6	Caffeic acid glucoside	341	179	135		**X**
7	Kaempferol rhamnoside *	431	285	-	**X**	
8	Quercetin rhamnoside **	447	301	179, 151	**X**	
9	Isorhamnetin xyloside *	447	315, 301, 131, 161, 285, 379	-	**X**	
10	Quercetin glucoside **	463	301	179, 151	**X**	**X**
11	Rutin	609	301	179, 151		**X**

*: Only detected by LC-MS. **: Detected by LC-MS and ID-MS.

**Table 2:** Antioxidant activity of extracts of the *Bursera microphylla* Magdalena ecotype.

**Extract**	**DPPH** **IC_50_ (μg/mL)**	**FRAP** **μ** **M** **Fe(II)/g** **dw**
Fruit	105.40 ± 0.48 ^a^	2034.3 ± 89.7 ^a^
Stem	52.90 ± 0.02 ^b^	1419.5 ± 115.3 ^b^

^a,b^ Different letters within the same column indicate significant statistical differences (*p* < 0.05). All values are expressed as mean ± standard deviation (SD). IC_50_: Concentration to inhibit 50% of DPPH radical. FRAP: Ferric reducing antioxidant power, based on the reduction of Fe(II)/g dw: Dry weight.

**Table 3:** Anti-inflammatory effect (IC_50_) of extracts of the *Bursera microphylla* Magdalena ecotype.

Extract	NO (μg/mL)	TNF-α (μg/mL)
Fruit	93.6 ± 4.6 ^a^	101.5 ± 9.1 ^a^
Stem	119.7 ± 2.8 ^b^	143.4 ± 4.9 ^b^

IC_50_: Concentration required to inhibit 50% of nitric oxide (NO) or TNF-α production. All values represent mean ± standard deviation (SD) of three independent experiments. ^a,b^ Values with different letters indicate significant statistical differences (*p* < 0.05).

**Table 4:** Antiproliferative effect (IC_50_) of fruit and stem extracts of the *Bursera microphylla* Magdalena ecotype.

Extract	LS180	C-33 A	ARPE-19
Fruit	0.7 ± 0.001 ^a^	0.6 ± 0.07 ^a^	77.9 ± 4.3 ^a^
Stem	2.2 ± 0.1 ^b^	0.7 ± 0.08 ^a^	85.2 ± 3.5 ^b^

IC_50_: Concentration required to inhibit 50% of cell proliferation. All values represent mean ± standard deviation (SD) of three independent experiments. ^a,b^ Values with different letters indicate significant statistical differences (*p* < 0.05).

**Table 5:** Selectivity index (SI) of fruit and stem extracts of the *Bursera microphylla* Magdalena ecotype against cancer cell lines.

Extract	LS180	C-33 A
Fruit	111.2	129.8
Stem	38.7	121.7

## Data Availability

Please add the corresponding content of this part.

## Supplementary Materials

The following supporting information can be downloaded at: https://www.mdpi.com/article/10.3390/plants14213357/s1, Figure S1: Full scans of ESI-IT-MS analysis of ethanolic extract from the stem of *B. microphylla*. Figure S2: Full scans of ESI-IT-MS analysis of ethanolic extract from the fruit of *B. microphylla*. Figure S3: *Bursera microphylla* A. Gray.
