# Supplementary material for: Biological Activities and Phenolic Profile of Bursera microphylla A. Gray: Study of the Magdalena Ecotype
Source: Plants (Basel). 2025 Nov 2;14(21):3357. doi: 10.3390/plants14213357 (PMC12608420; doi:10.3390/plants14213357)
Supplement: Supplementary file 1 [file plants-14-03357-s001.zip › plants-3894554-supplementary.pdf]

Supplementary Material
Biological Activities and Phenolic Profile of *Bursera microphylla* A. Gray: Study of the Magdalena Ecotype

Heriberto Torres-Moreno*, Julio César López-Romero*, Max Vidal-Gutiérrez, Karen Lillian Rodríguez-Martínez, Ramón E. Robles Zepeda, Wagner Vilegas, Ailyn Oros-Morales

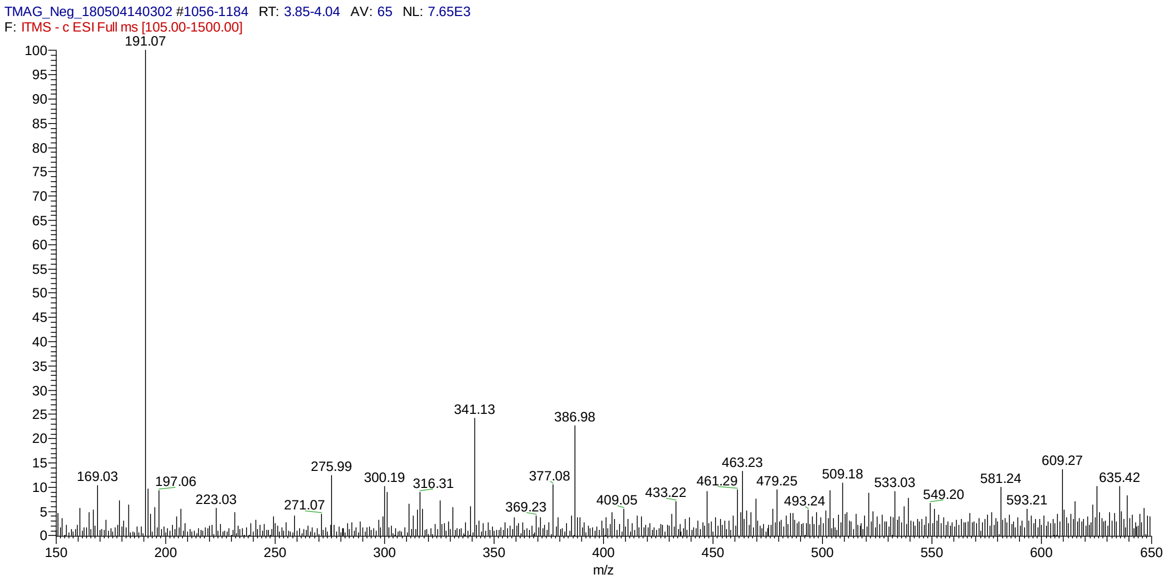

Figure S1. Full scans of ESI-IT-MS analysis of ethanolic extract from the stem of *B. microphylla*.

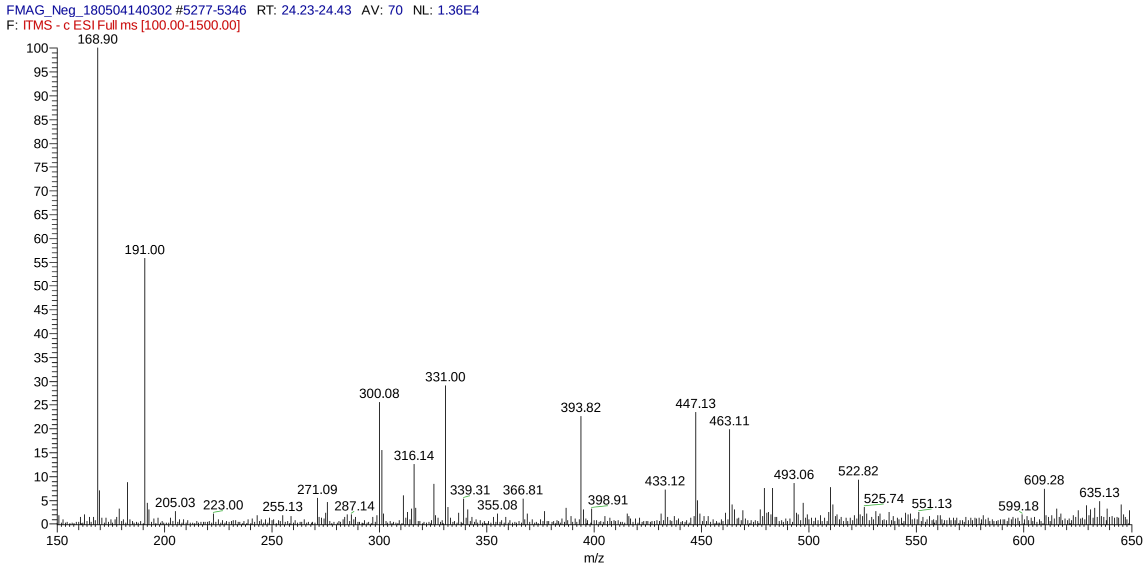

Figure S2. Full scans of ESI-IT-MS analysis of ethanolic extract from the fruit of *B. microphylla*.

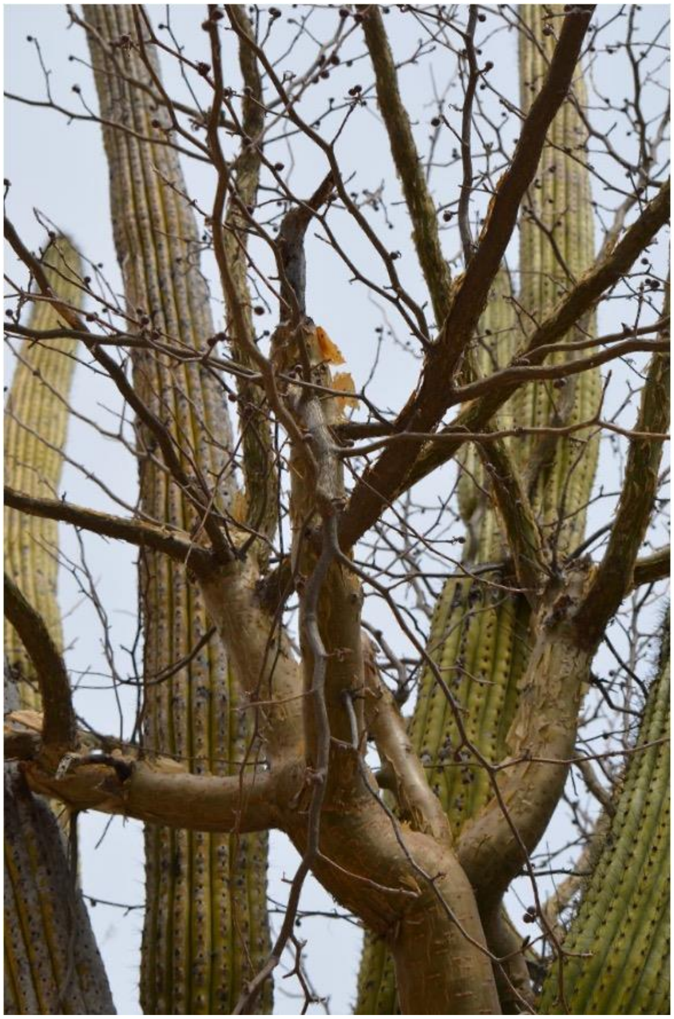

Figure S3. *Bursera microphylla* A. Gray.
